# Three-part proximal humerus fracture: a full-percutaneous severely displaced greater tuberosity reduction and intramedullary nailing technique

**DOI:** 10.1016/j.xrrt.2025.05.016

**Published:** 2025-06-09

**Authors:** Edoardo Giovannetti de Sanctis, Bastien Bige, Jonathan Cornacchini, Nicolas Bronsard, Jean-François Gonzalez, Marc-Olivier Gauci

**Affiliations:** IULS- Institut Universitaire Locomoteur et Sports, Pasteur 2 Hospital, CHU, Nice, France

**Keywords:** Proximal humerus fracture, Intramedullary nailing, IMN, PHF, Neviaser, Nail

The ideal surgical management of proximal humeral fractures (PHFs) remains controversial.^4^^,^^10^^,^^15^ Locking plates have been considered traditionally the “gold standard” treatment for these fractures. However, their use has been shown to be associated to a significant high rate of complications, leading to the development of humeral intramedullary nails (IMNs) as an attractive alternative treatment. Third-generation straight IMNs have been shown to have biomechanical advantages: with multiplanar screws at different levels and directions.^2^^,^^12^^,^^14^

During the past decade, although the great variations in nail designs (including both first-generation and more modern straight nails), fracture type and surgical approach, literature supports the use of IMN for PHFs, showing a lower complication rate.^3^^,^^11^

The use of a percutaneous technique for the IMN of displaced 2-part surgical neck fracture has increased significantly in the last decade.^3^^,^^9^, ^10^, ^11^

However, in case of a severely displaced 3-part PHF an open approach by an extended anterolateral or superior incision is still considered the gold standard to reduce the greater tuberosity (GT) with traction sutures, as the primary goal is to reach an anatomic consolidation of the fragments. GT optimal reduction is the key point in PHFs treatment, influencing the risk of head avascular necrosis (AVN).^5^^,^^7^^,^^8^

However, the notable benefits of performing minimal invasive surgery include less postoperative pain, fewer complications, improved cosmetic results with less scarring, shortened hospital stay and faster recovery time.

An open approach might increase the risk of humeral head AVN, altering the blood supply to the humeral head.^16^

The objective of the present article is to describe a novel technique to treat all displaced 3 parts PHFs with a full-percutaneous GT indirect reduction and IMN.

The main goal was to create an effective and safe surgical procedure allowing proximal humeral fragments reduction with limited tissue dissection.

## Surgical technique

### Indications and preoperative planning

This technique is indicated in case of 3-part PHFs with displaced GT and humeral head fragments (Table I).^13^

**Table I:** Surgical indications.

3 parts PHF
Highly displaced greater tuberosity and proximal humeral head
Absence of glenohumeral osteoarthritis or humeral head split

*PHF*, proximal humeral fracture.

**Figure 1:**
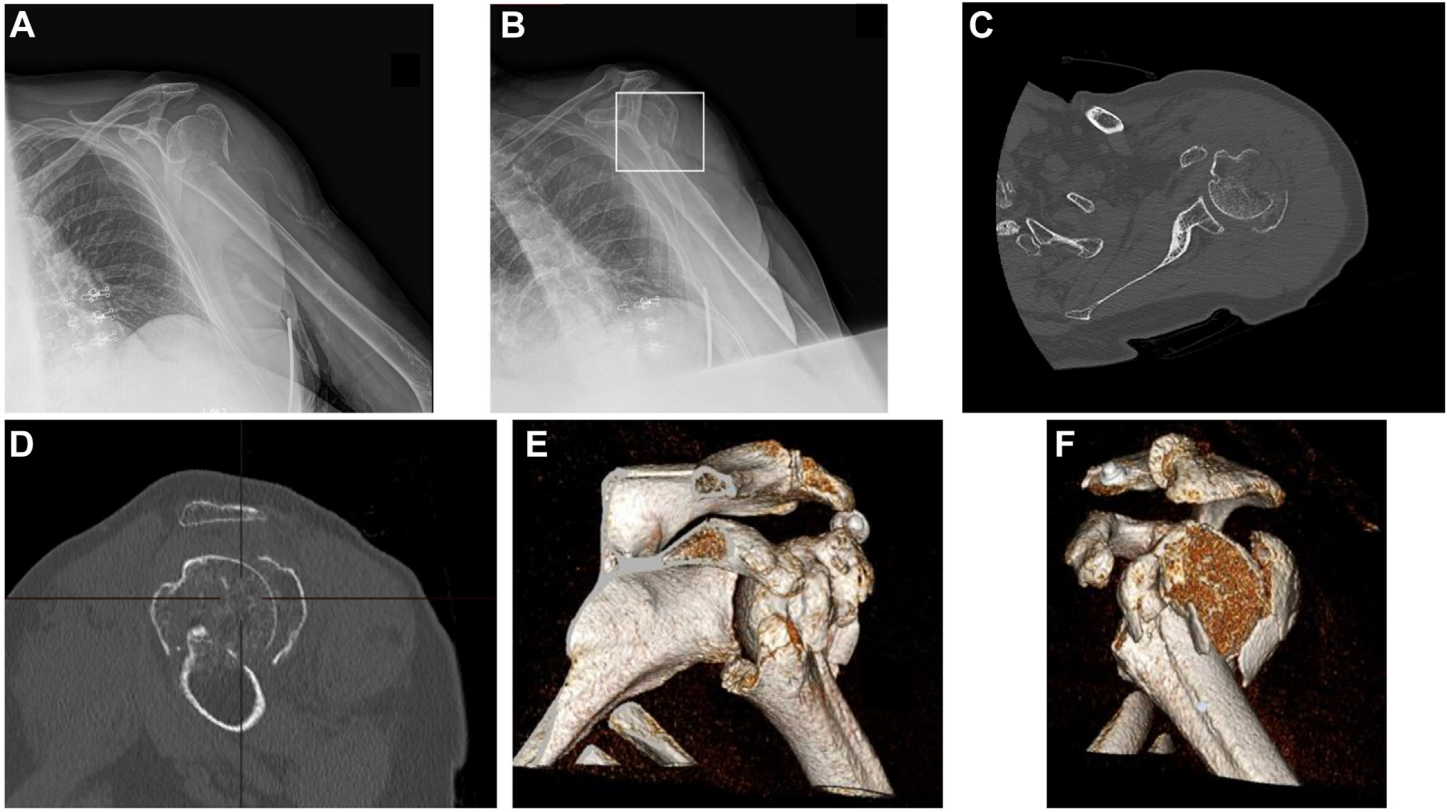
A 3-part fracture of the proximal humerus with a posteromedially displaced greater tuberosity evaluated through the radiographs (**A** and **B**), the CT axial (**C**) and sagittal (**D**) plane and 3-dimensional CT reconstruction (**E** and **F**). *CT*, computed tomography.

All PHFs are evaluated with both a true anteroposterior (AP) and a lateral view radiograph and a 3-dimensional computed tomography scan (Fig. 1).

The 3-part PHFs show frequently the same fragment displacement due to the muscle deforming forces. The GT is typically displaced posteromedially due to the rotator cuff traction whereas the main vertical fracture plane is typically posterior to the bicipital groove.

### Step 1: patient positioning: lazy beach chair

**Figure 2:**
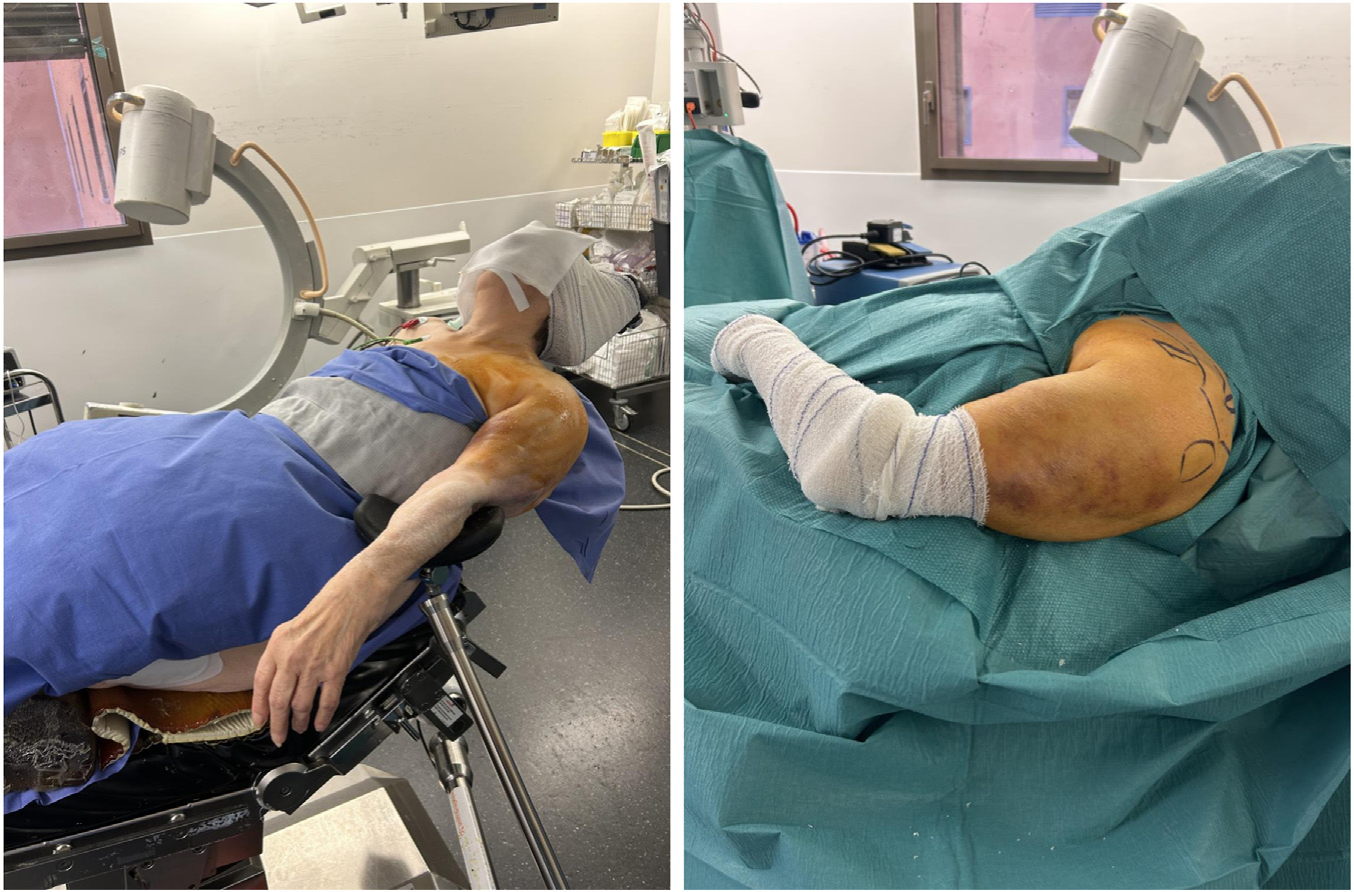
Patient positioning: lazy beach chair. A pneumatic arm holder (Spider; Smith & Nephew, Wixom, MA, USA) is used to help the surgeon in positioning the arm. Left: Before.

The patient, under *general anesthesia* combined with interscalene block, is placed in a lazy beach chair position on a table without the posterior shoulder supporting pads or on a radiolucent table (Fig. 2).

A pneumatic arm holder (Spider; Smith & Nephew, Andover, MA, USA) is used to help the surgeon in positioning the arm of the patient in the desired position during the whole surgery. The arm is always held in neutral rotation.

The image intensifier is usually placed parallel to the longitudinal axis of the patient. Two orthogonal radiographic views (the AP and lateral view) are obtained by tilting the C-arm medially or laterally.

**Figure 3:**
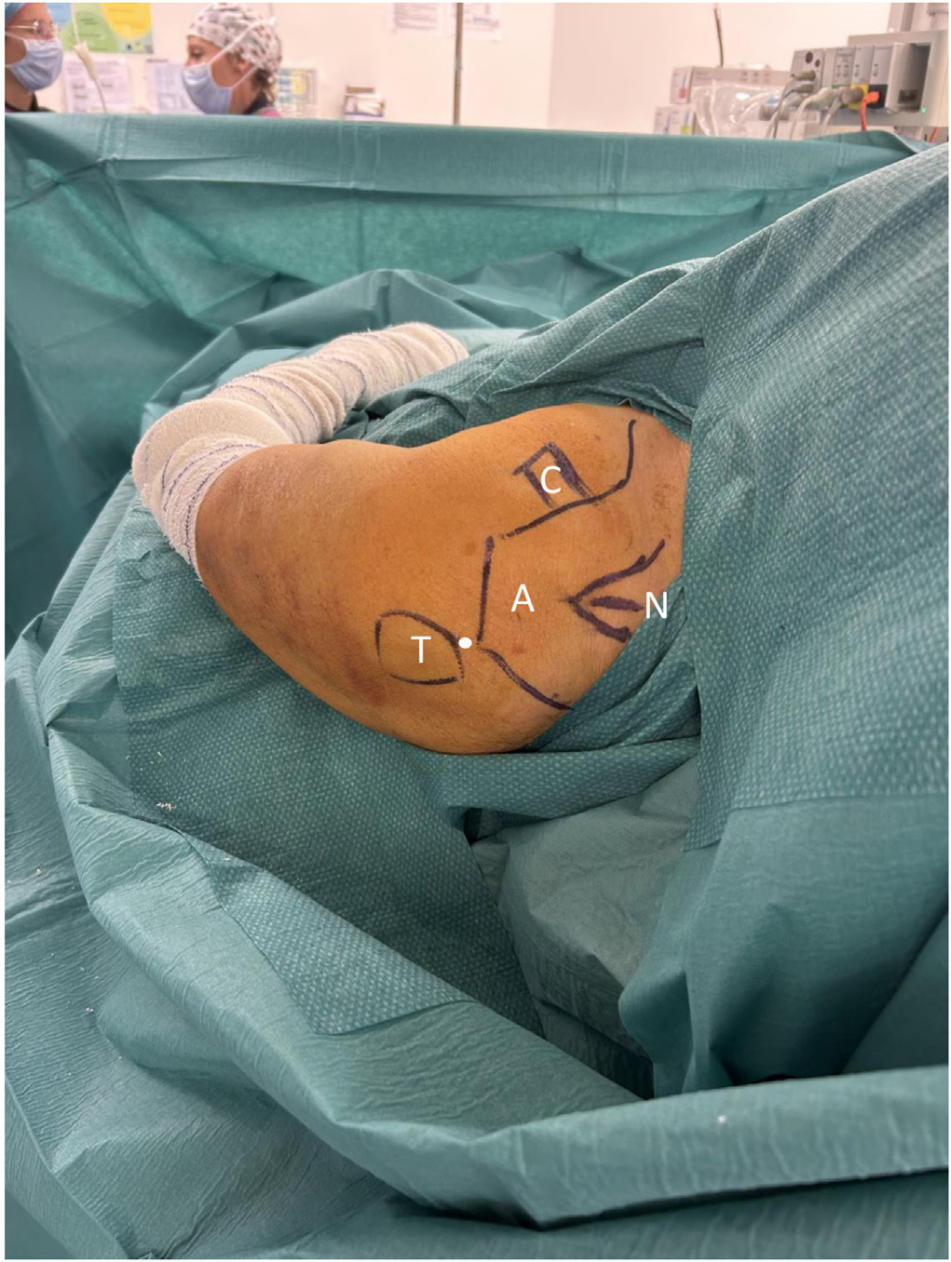
The preoperative anatomical landmarks and surgical approach. The *white circle* represents the K-wire entrance. *C*, coracoid; *A*, acromion; *N*, Neviaser; *T*, greater tuberosity.

Preoperative cleansing with povidone-iodine surgical scrub followed by povidone-iodine antiseptic solution is performed before surgery. The patient is draped and the skin anatomical landmarks are marked (Figs. 2 and 3).

### Step 2: surgical neck fracture reduction

**Figure 4:**
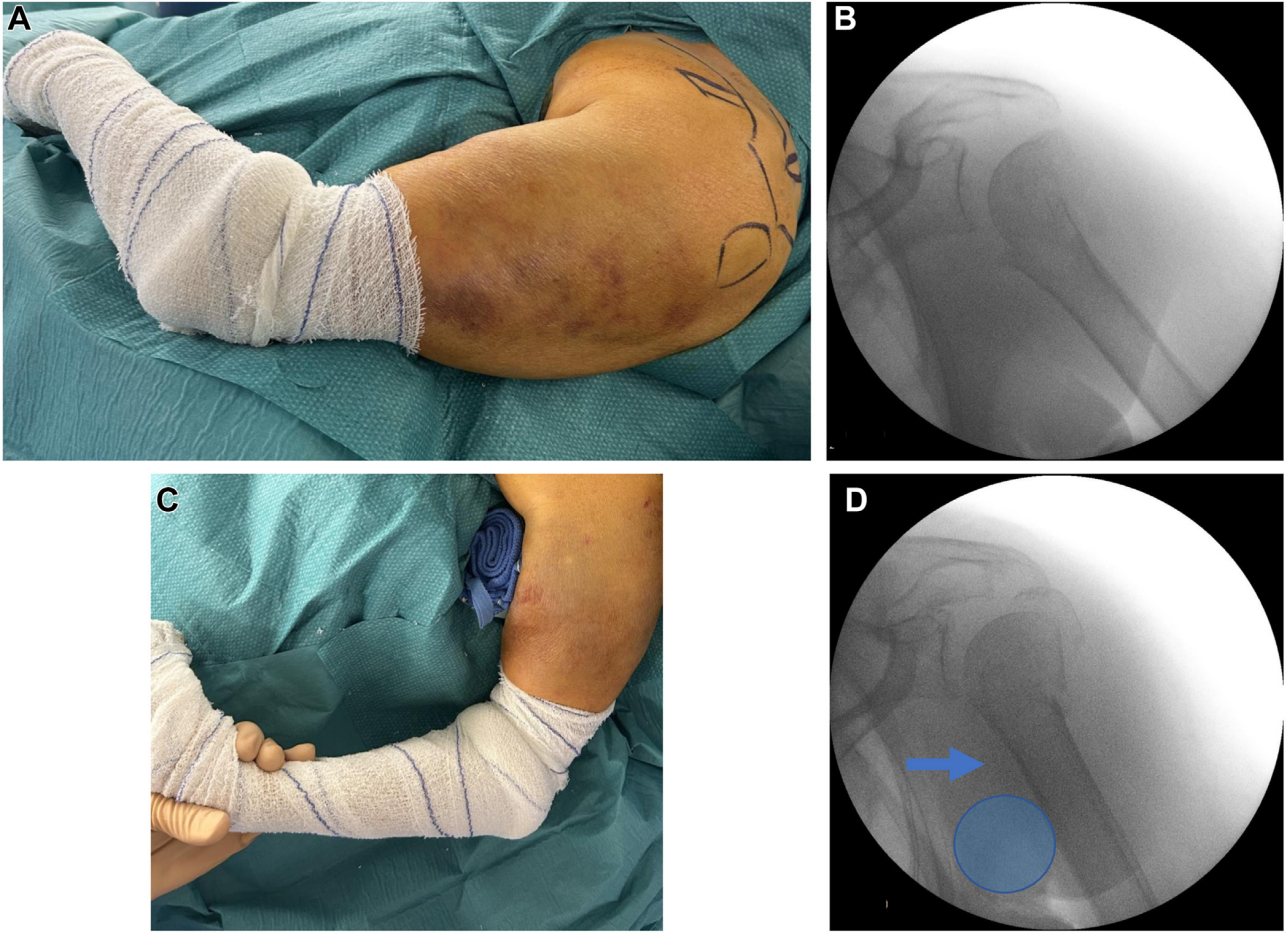
Surgical neck fracture reduction through fluoroscopic control (**B**-**D**) placing a surgical gauze roll into the axillary cavity (**A**-**C**). *Blue circle* represents surgical gauze roll; and *blue arrow* represents force direction produced by the surgical gauze roll.

The surgical neck fracture is first reduced by placing the arm in neutral rotation and by applying a surgical gauze roll into the axillary cavity (Fig. 4).

### Step 3: greater tuberosity and humeral head reduction

A Steinmann pin or a K-wire is introduced percutaneously (without a stab incision) under fluoroscopic visualization to reduce and hold the GT and humeral head fragments in place before nail and screws insertion. The pin skin introduction should follow the displacement of the GT assessed preoperatively on the computed tomography scan. It is frequently at the level of the posterolateral corner or the acromion (Fig. 3).

**Figure 5:**
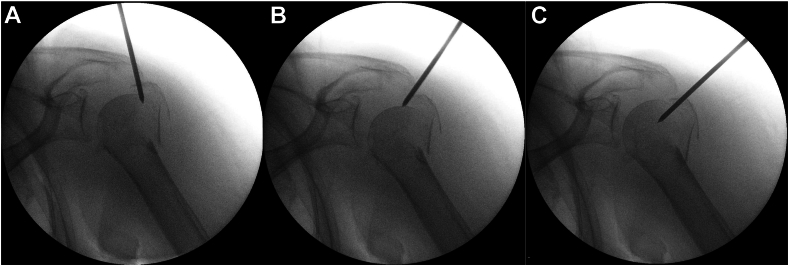
GT and humeral head fragment Reduction. A K-wire is used as a “joystick” and place between the 2 fragments (**A**). The aim is reducing inferolaterally the GT while simultaneously lifting the humeral head if displaced in valgus (**B**-**C**). *GT*, greater tuberosity.

It is inserted through the fracture between the 2 fragments and used as a “joystick” with the aim of reducing inferolaterally the GT while simultaneously lifting the humeral head if displaced in valgus (Fig. 5). It therefore manipulates the 2 fragments in a more anatomic position previous and during nail insertion.

### Step 4: nail insertion

**Figure 6:**
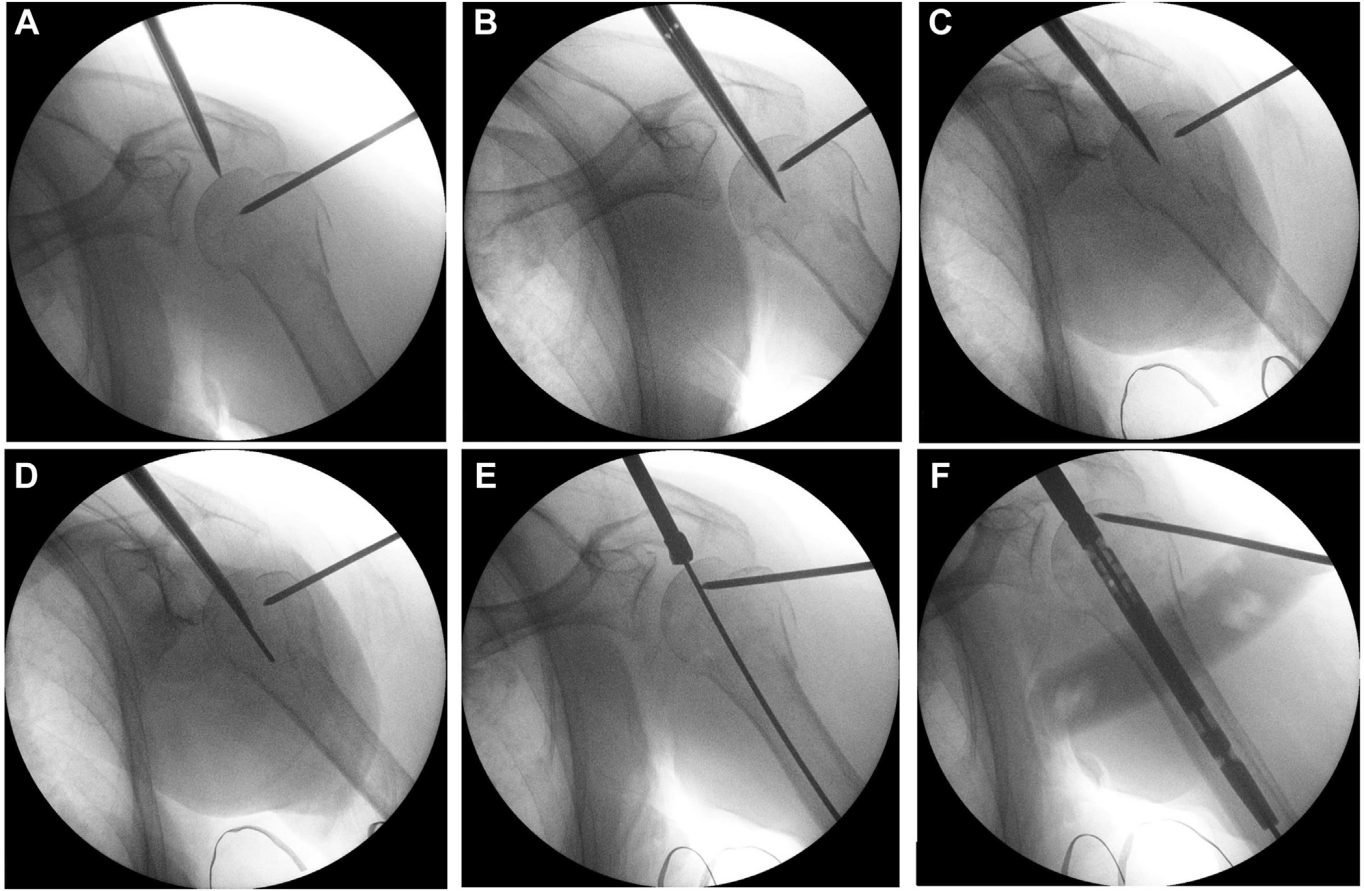
A cannulated starter awl is inserted through the supraspinatus muscle (**A**-**C**). Insertion of a guidewire (**D**) and a 9.5 mm cannulated reamer over the guidewire (**E**). The humeral nail is then manually inserted along the guide wire (**F**).

The Neviaser portal approach is used (Fig. 3). A 2 cm stab incision is made posteriorly to the acromioclavicular joint. A longitudinal supraspinatus split in the line of the tendon fibers is made with curved scissors at the musculotendinous junction to gain access to the humeral head. A cannulated starter awl is inserted through the supraspinatus muscle into the humeral head and its position is confirmed through fluoroscopic control (Fig. 6, *A*). The correct entry point is situated at the apex of the humeral head in line with the axis of the humeral shaft, in both AP and lateral view (Fig. 6, *B* and *C*). A guide wire is then inserted and advanced into the medullary canal (Fig. 6, *D*).

A 9.5 mm cannulated drill bit reamer is passed over the guide wire and used to prepare the humeral head before nail insertion (Fig. 6, *E*).

The 8 mm × 130 mm anterograde Tornier AEQUALIS IM Nail (Stryker, Kalamazoo, MI, USA) is then mounted on an insertion handle. The extension for the lesser tuberosity (LT) screw is then assembled to the external device.

The Humeral nail is then manually inserted along the guide wire, using gentle and slight rotating movements until proper depth is reached, which is between 5 and 10 mm from the humeral head subchondral bone (Fig. 6, *F*). Make sure the nail enters the distal fragment properly.

Confirm through fluoroscopy the top of the nail is fully buried beneath the bony surface of the humeral head, as no protrusion of the nail may be tolerated. Once the nail is placed within the humeral canal, the guide wire is removed.

### Step 5: anterior proximal screw insertion

**Figure 7:**
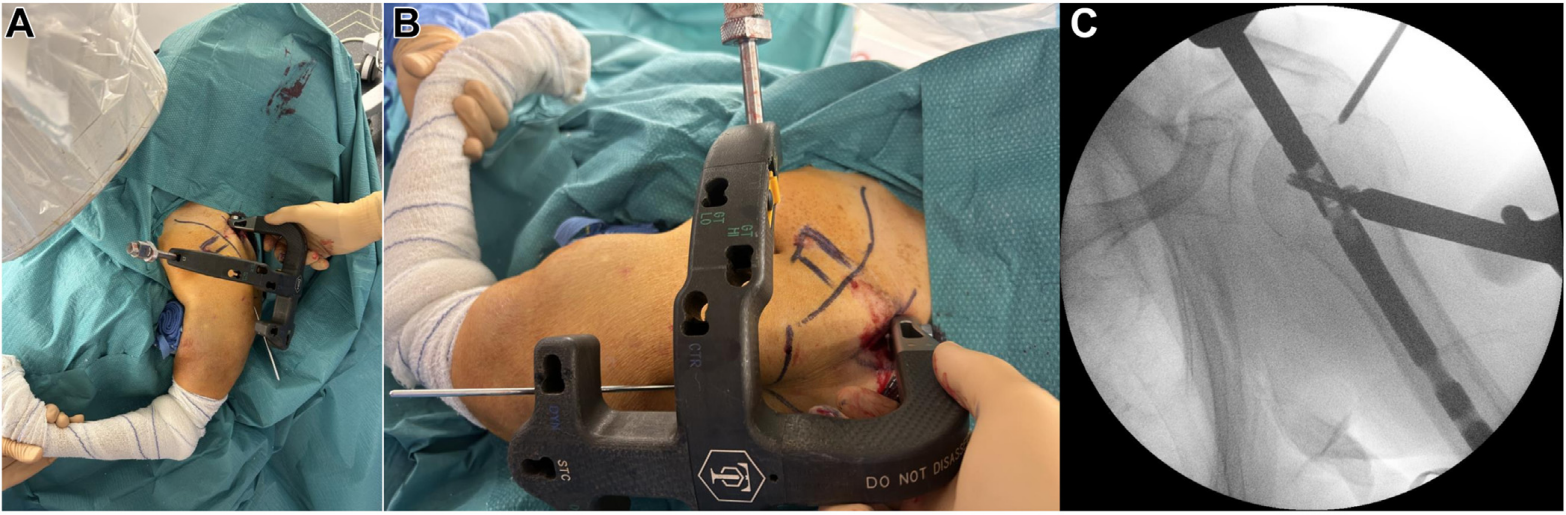
Through the LT screw anterior extension, the screw sleeve with the drill guide is inserted with the arm in adduction and neutral rotation and the version rod in slight internal rotation (**A** and **B**). The drill guide is then removed and the anterior screw is inserted into the LT (**C**). *LT*, lesser tuberosity.

Through the LT screw anterior extension, the screw sleeve with the drill guide is inserted with the arm in adduction and neutral rotation and the version rod in slight internal rotation (Fig. 7, *A* and *B*).

After a 1 cm stab incision, blunt dissection down to the bone is performed to place the screw sleeve in contact with the LT bone without interposition of soft tissue. A 3.5 mm diameter drill bit is inserted into the drill guide. It is mandatory not to penetrate the second cortex. The appropriate 5 mm diameter proximal locking screws length might be measured directly. However we normally use 36 and 32 mm in length respectively for males and females for each of the proximal screw. The proximal locking screws length should then be then fluoroscopically verified. The drill guide is then removed and the anterior screw is inserted into the LT (Fig. 7, *C*).

To ensure that the inserted screw is positioned within the nail hole, the guide wire previously used is inserted again within the cannulated nail. The distal tip of the guide wire should be blocked by the inserted screw and not be allowed to get further inferiorly through the nail canal.

The guide wire portion at the level of the skin incision is marked with a Kocher. Once confirmed the right positioning of the screw, the guide wire with the Kocher still attached might be removed from the nail canal.

### Step 6: greater tuberosity reduction

**Figure 8:**
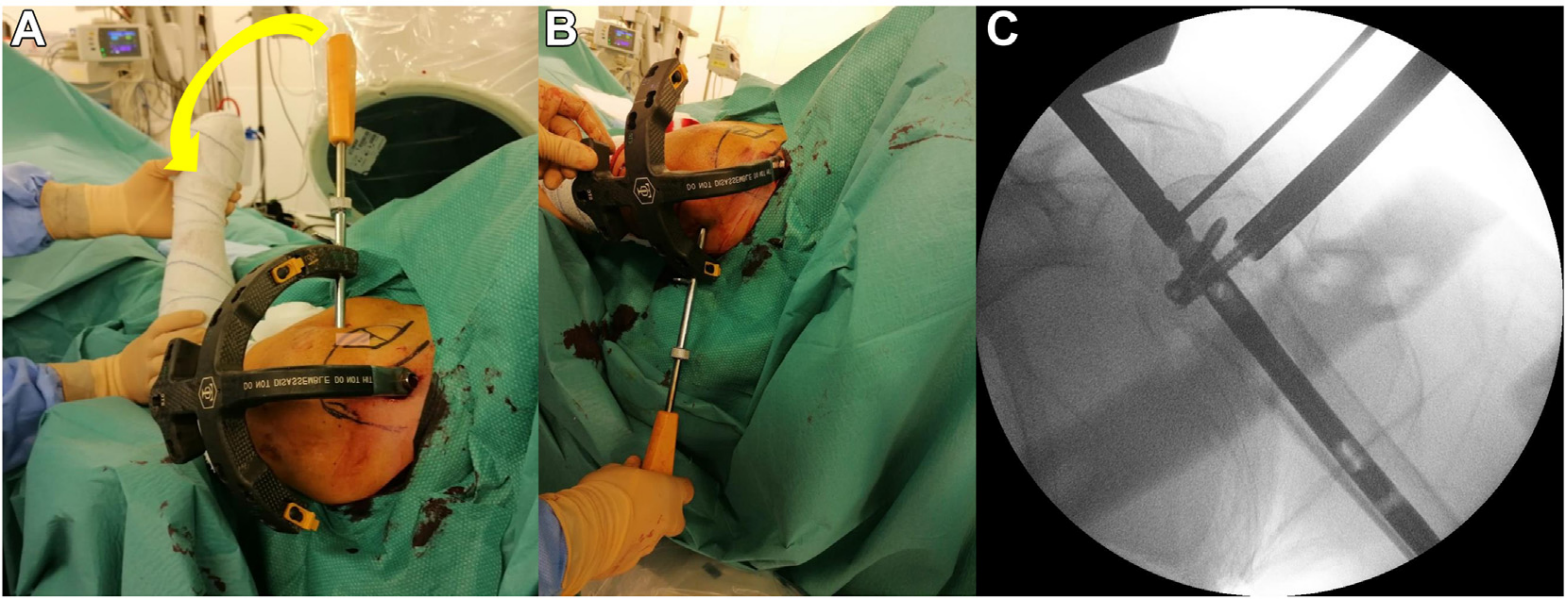
The lesser tuberosity is fixed to the humeral head and the nail and the external device is rotate in external rotation while maintaining the arm in neutral rotation (**A**). Two posterior proximal locking screws are inserted within the GT, from posterolateral to anteromedial (**B**). The first GT locking screw to be inserted is the most inferior (**C**). *GT*, greater tuberosity.

After placing the first screw, the LT is fixed to the humeral head and the nail. The screw sleeve and the screw driver are held in place and are used to rotate the rod and therefore the nail in external rotation while maintaining the arm in neutral rotation (Fig. 8, *A*). This closed maneuver will reduce the GT fragment to the humeral head.

### Step 7: posterior proximal screws insertion and final surgical neck reduction

When a GT anatomical reduction is achieved, 2 posterior proximal locking screws are inserted within the GT, from posterolateral to anteromedial (Fig. 8, *B*). The steps for screw insertion described previously are repeated for the posterior proximal locking screws.

The first GT locking screw to be inserted is the most inferior (Fig. 8, *C*). With this implant design, the anterior screw hole is between the 2 posterior screw holes. Therefore the technique previously described to check whether the screw is inserted into the screw hole and not adjacent to the nail, might not be used for the most inferior posterior proximal screw, as it is below the anterior locking screw. In this case, the right positioning of the screw into the proper hole should be confirmed through fluoroscopy.

**Figure 9:**
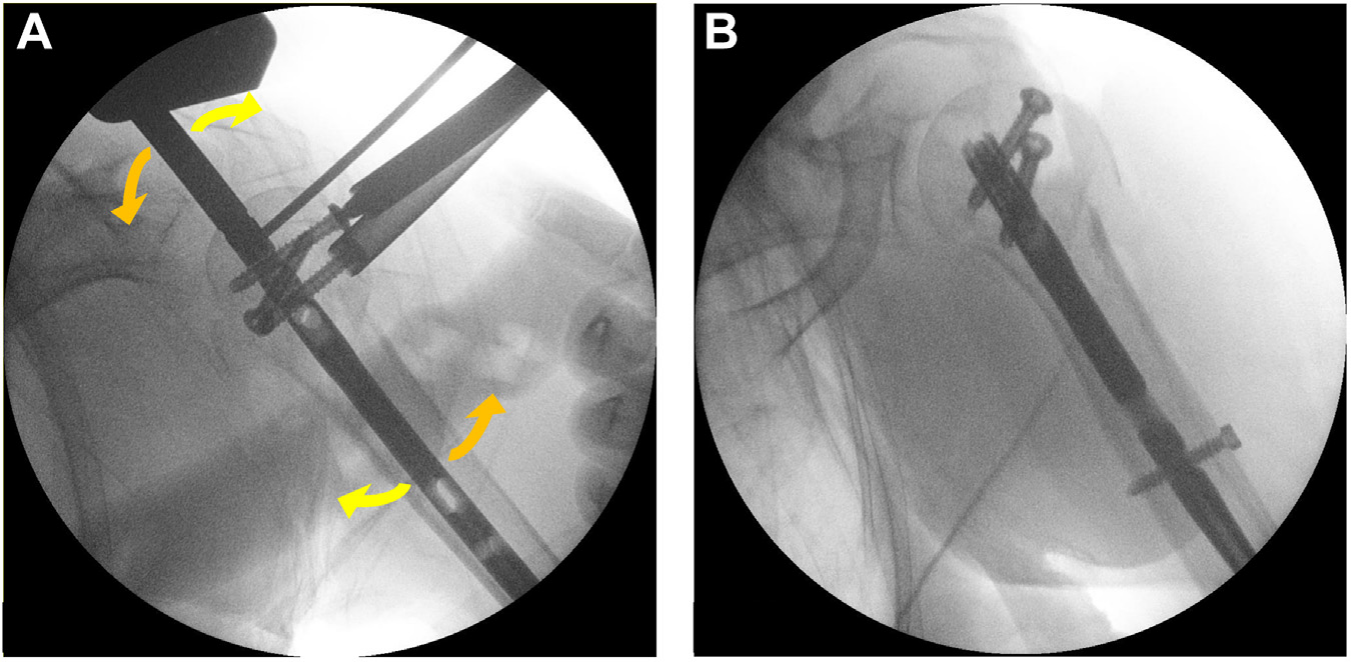
The second GT locking screw is inserted (**A**). First distal screw insertion (**B**). *GT*, greater tuberosity.

The second GT locking screw is then inserted (Fig. 9, *A*). Its hole is positioned above the anterior locking screw hole. The guide wire, with the Kocher still attached, previously used is inserted again into the cannulated nail. If the most superior GT screw is inserted into its screw hole, the guide wire is not allowed to get further deep within the canal and the distance between the Kocher marked portion and the skin incision is increased. The guide wire is then removed. Fluoroscopic control can then be used to ensure the proper length and position of all the screws.

### Step 8: final surgical neck reduction and distal screw insertion

A final reduction of the head fragment and the humeral diaphysis at the surgical neck is performed on both the axial and coronal plane by using the nail as a joystick.

To reduce the surgical neck fracture on the axial plane, internally rotate to neutral position the nail using the GT screwdriver left in place into the external device, (which has been previously placed in external rotation to reduce the GT), to recreate the physiological retrotorsion of the humeral head.

Furthermore the nail might be used to reduce the humeral head and diaphysis on the coronal plane, while placing it in valgus or varus stress (Fig. 9, *A*).

### Step 9: distal screw insertion

Once proper reduction is achieved, the nail is fixed distally with 1 or 2 distal screws. The second screw is recommended especially in osteoporotic bone.

The distal screw positioning follow the same steps of proximal locking screws: screw sleeve and drill guide positioning through the external targeting device, 1 cm stab incision and soft tissue blunt dissection down to the bone, advancement of the screw sleeve and drill guide in against the bone, bicortical hole drilling with a 3.5 mm drill bit, fluoroscopic control to define the appropriate screw length while drilling (which is normally between 22 and 26 mm) and screw insertion. The same steps are followed to place a second distal screw.

Once all bone fragments are secured to the nail, the external targeting device is detached from the nail.

**Figure 10:**
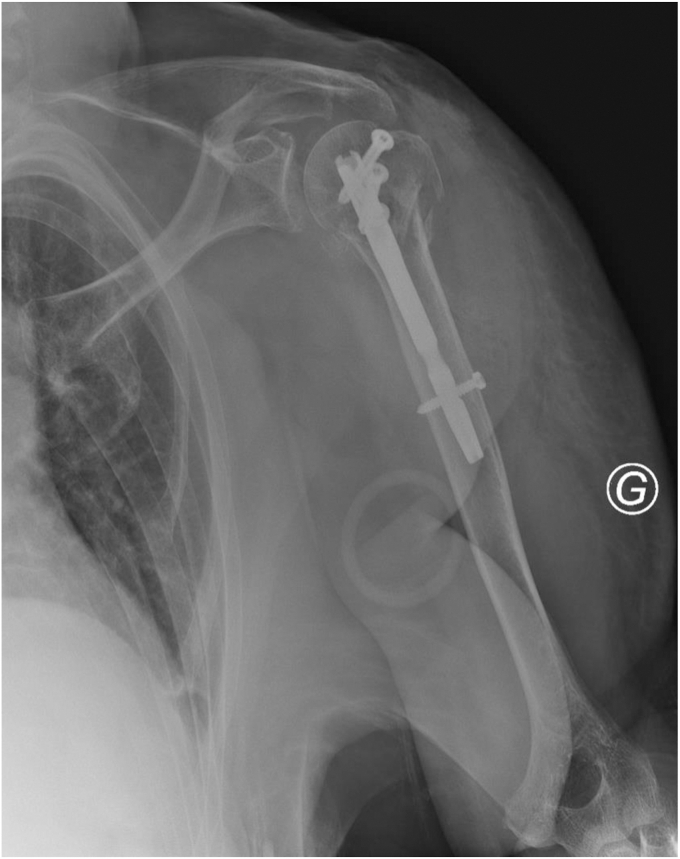
Radiographic assessment after closure.

**Figure 11:**
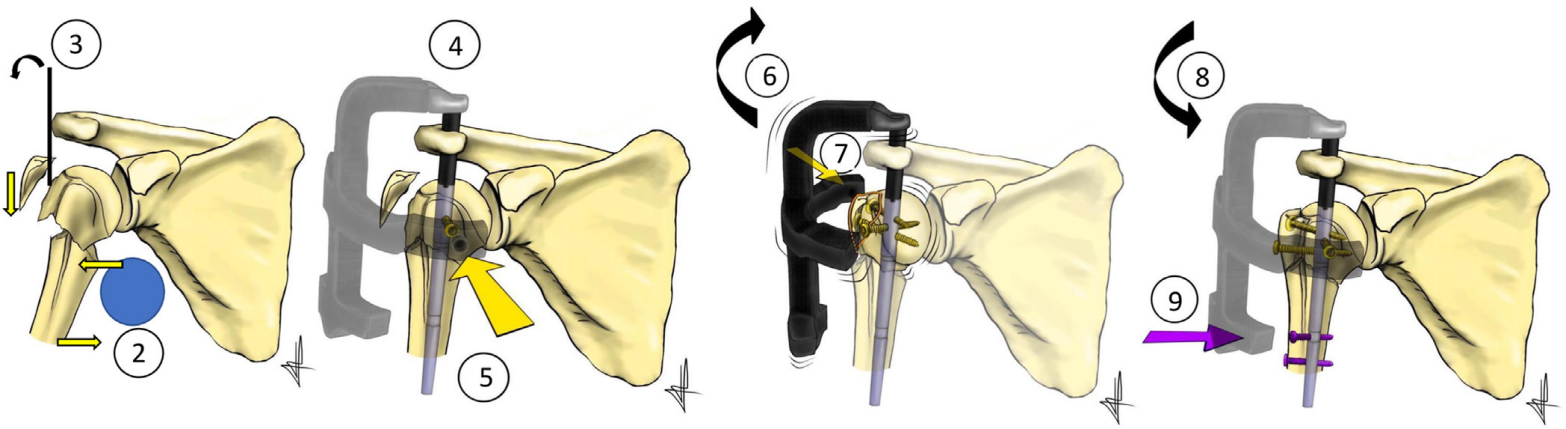
Schematic drawing of the surgical technique. Technique's steps (2-9) mentioned and numbered in Table II.

Subcuticular skin closure with absorbable Vicryl No. 2.0 and skin closure with *Steri*-*Strips* (*3M*, St. Paul, MN, USA). Radiographic assessment after closure (Fig. 10). The surgical technique is summarized in Table II and Figure 11.

**Table II:** Step-by-step details of the technique.

Step 1. Patient positioning: Lazy beach chair
Table without the posterior shoulder supporting pads or on a radiolucent table.
The arm is always held in neutral rotation
Step 2. Surgical neck fracture reduction
Application of a surgical gauze roll into the axillary cavity
Step 3. GT and humeral head reduction
Percutaneous introduction of a K-wire at the level of the posterolateral corner or the acromion
It is used as a “joystick” to reducing inferolaterally the GT and lifting the humeral head
Step 4. Nail Insertion
The Neviaser portal approach is used
A longitudinal supraspinatus split in the line of the tendon fibers is made
The correct entry point is situated at the apex of the humeral head in line with the axis of the humeral shaft
A guide wire is then inserted and advanced into the medullary
A 9.5 mm cannulated drill bit reamer is passed over the guide wire
The Humeral nail is then manually inserted along the guide wire
Step 5. Anterior proximal screw insertion and GT reduction
The anterior screw is inserted into the lesser tuberosity
We normally use 36 and 32 mm in length respectively for males and females for each of the proximal screw
Step 6. GT Reduction
Rotate the nail in external rotation while maintaining the arm in neutral rotation.
Step 7. Posterior proximal screws insertion and final surgical neck reduction
Two posterior proximal locking screws are inserted within the GT
Fluoroscopic control to ensure the proper length and position of all the screws
Step 8. Final surgical neck reduction and distal screw insertion
Internally rotation of the nail until neutral rotation to recreate the physiological retrotorsion of the humeral head
Reduce the humeral head and diaphysis on the coronal plane (valgus or varus stress)
Step 9. Distal screw insertion
The nail is fixed distally with 1 or 2 distal screws

*GT*, greater tuberosity.

### Postoperative management

**Figure 12:**
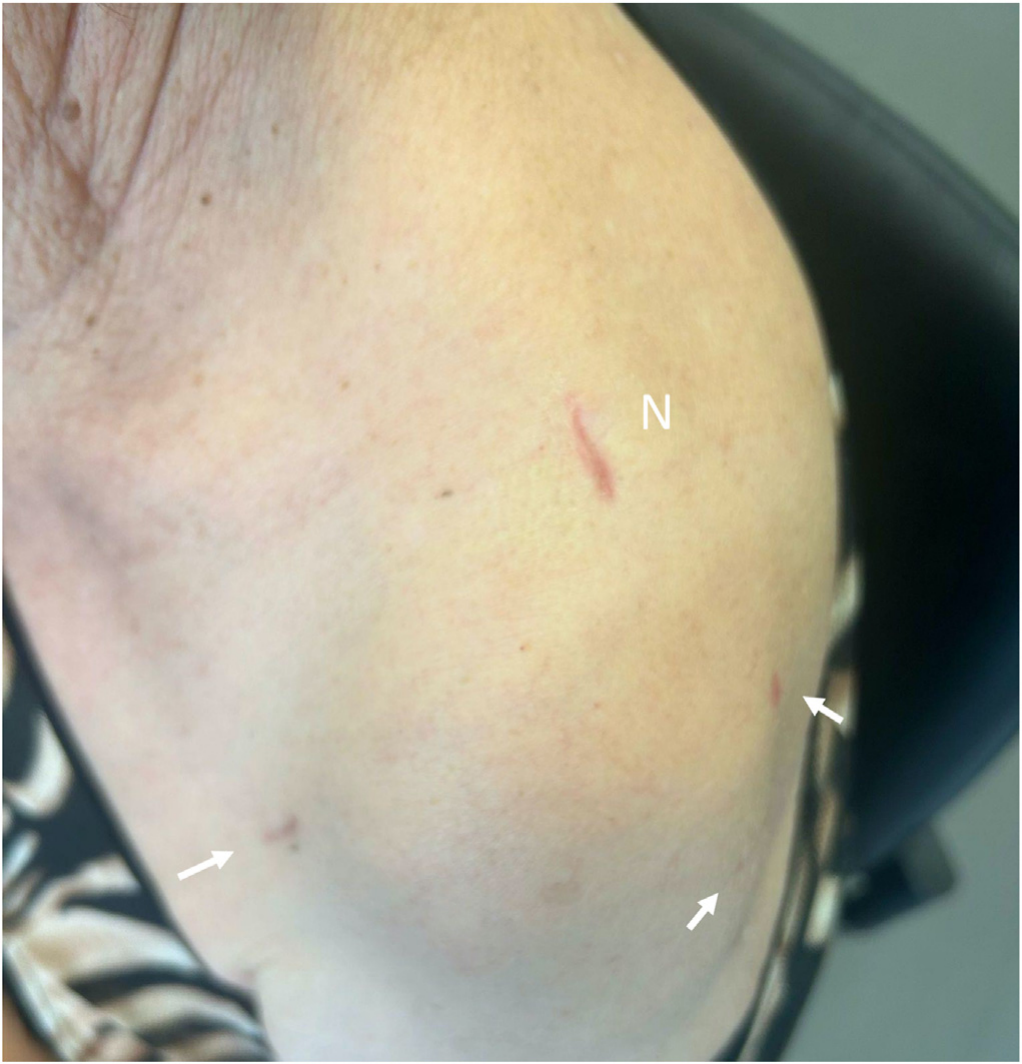
This technique is performed through very small incisions. *White Arrows* represent proximal screw skin incision. *N*, Neviaser.

A sling in neutral rotation is used for 3 weeks with immediate postoperative self-rehabilitation protocol involving gradual recovery of passive range of motion through self-stretching exercises. After 3 weeks, once the surgical incisions are healed (Fig. 12), Aquatic-Based Rehabilitation is started.

## Discussion

Most modern nails have been designed to avoid the complications described previously with first- and second-generation IMN.^1^^,^^14^ The third-generation design have several theoretical advantages.^1^ The smaller diameter enables to pass it through the muscular part of the supraspinatus. It uses locking and divergent tuberosities-oriented screws allowing for GT fixation without a greater risk of glenoid erosion in case of head necrosis and screws penetrating the humeral head.^4^^,^^8^^,^^11^

Furthermore, the straight design, having a more medial entry point, enables to increase the distance from the lateral head fracture line, leaving a bone ring which provides better fixation preventing secondary displacement, as the bone density is greater in the region of the superior humeral head compared to the GT.^2^

It is very important to insert an IMN through a correct entry point. The nail insertion through the traditional anterolateral approach may increase the risk of a rotator cuff tendon iatrogenic damage. It has been shown that an anterograde humeral nail might be inserted percutaneously via the Neviaser portal.^17^ It is a feasible and safe medial approach, which enables a more easily nail introduction through the highly vascularized supraspinatus muscle fibers, as the humeral head is usually displaced in varus and to reach the correct humeral head entry point with a symmetric bone ring around the nail (Table III).^6^

**Table III:** Advantages.

1. IMN
Decreased number of screws in the humeral head
Multiple plane of fixation (antirotational), with screws perpendicular to the fracture
Proximal nail tip fixation point
Shorten lever arm
Decreased risk of axillary nerve lesion at the level of the deep deltoid fascia
Reduced direct cost
Decreased complication and reoperation/revision rate
2. Neviaser portal (Medial approach)
Nail introduction through the highly vascularized supraspinatus muscle fibers
Correct entry point reached (with a symmetric bone ring around the nail)
Lowered risk of head varus malalignement
3. Percutaneous Approach
Lowered risk of axillary nerve damage
Less soft tissue dissection/disruption and preservation of blood supply
Shorter operative time

*IMN*, intramedullary nail.

At our department all PHFs (from 2- to 4- parts fractures) with an osteosynthesis indication are treated with IMN through the Neviaser portal. The choice between full-percutaneous and an open approach was previously based on the number of fragments and the amount of displacement of the greater and lesser tuberosities. The percutaneous approach was preferred for the treatment of displaced 2-part PHFs or PHFs with no displacement of the GT or LT. However, in case of displaced GT and LT PHFs, the primary goal is to reach an anatomic consolidation of the fragments; therefore, in these cases an open reduction of the fragments was indicated and performed through a superior approach. Tuberosity malreduction leads to poor clinical outcomes, due to retraction and atrophy of the posterosuperior rotator cuff with an increased risk of AVN.^11^

However, open surgery increases the rate of postoperative infection, nerve injury and hematoma and it is associated with greater tissue damage with scar and tissue adhesions, a longer healing period and improper cosmetic outcomes.

The goal of the described technique for performing a full-percutaneous GT reduction and IMN was to achieve a stable fixation of a 3-part PHF with minimum soft tissue disruption.

The IMN might be performed either with a lazy beach chair and a lateral position. The lazy beach chair enables a conversion to an open approach if the GT reduction is not achieved through a percutaneous technique.

The surgical approach used at our institution with the PHF intramedullary nailing varies based on the fracture pattern and fragment displacement. PHFs with no GT fracture or displacement were normally treated with a full-percutaneous technique in a lateral position, whereas severely displaced 3- or 4-part PHSs are performed through an open approach in a lazy beach chair position. To date however, no case treated so far with the described technique has then been converted to an open approach. Therefore, once confirmed with a greater number of patients, the lateral position might be preferred, due to the easiness of performing the 2 orthogonal radiographic views with the image intensifier.

The reduction of the fragments is performed through several steps, with the aim of counteracting the deforming forces on the proximal humerus in the setting of a 3-part PHF.

Part of the reduction is performed before incision using a surgical gauze roll. The pectoralis major, the teres major and the latissimus dorsi applies an anteromedial force onto the proximal part of humeral shaft (below the surgical neck). The deltoid applies a superiorly directed force on the diaphysis of the humerus below the Pectoralis major insertion. These 2 muscles provide a valgus deforming force with abduction of the shaft fragment. The surgical gauze roll reduces the proximal part of the humeral diaphysis, adducting the shaft fragment.

Part of the reduction is then performed before inserting the IMN, while using a K-wire as a “joystick” with the aim of reducing inferolaterally the GT. The K-wire is used to counteract the deforming forces produced by the supraspinatus, the infraspinatus, and teres minor which pull the GT posteromedially.

The K-wire might be used also to lift simultaneously the humeral head if displaced in valgus. As the Neviaser portal is medial to the cartilage entry point, the nail is inserted with a lateral inclination within the humeral head.^9^ The inclination is then reduced to insert the nail through the proximal diaphysis (with a valgus head reduction effect), lowering the risk of head varus malalignement, which is worse tolerated than valgus impacted fractures, increasing the risk of postoperative loss of reduction.^4^ However, if a preoperative valgus displaced humeral head is not reduced before positioning the nail, the valgus displacement might increase with IMN insertion through a medial portal.

A precise nail entry point and height is crucial to avoid respectively further fracture displacement and both acromial impingement or axillary nerve damage.

It is mandatory to avoid placing the arm in internal rotation after this procedure, as it might place the GT under tension of the RC and potentially increase the risk of humeral head fragment malrotation.

No study to date has reported the functional outcomes of a severely displaced GT 3 part PHF treated with a full-percutaneous IMN.

Described complications of PHFs treated through an IMN include screw penetration into the joint, backed out screw, superior protrusion of the nail with acromial impingement, infection, AVN, nonunion or malunion, loss of reduction, secondary cuff tear, GT reabsorption.^8^^,^^12^

Advantages of a percutaneous approach are: less bleeding, less invasive surgery, close reduction of the fracture, less vascular nerve injuries, shorter time of surgery, better esthetic results (Table III) (Fig. 12).

The disadvantages of this technique might be: imperfect fragments reduction and increased exposure to X-rays.

Historically, at our institution the surgical approach to be used in case of a 3 parts PHF treated with an IMN was chosen based on surgeon's preference and GT involvement and displacement. A percutaneous approach was indicated in case of fractures without tuberosity involvement or with no GT displacement.^11^ When the tuberosities were involved a superior-transdeltoid approach was preferred to facilitate the reduction of the GT fragment.

Since the development of the described surgical technique each 3-part PHF has been treated with a full-percutaneous approach. However, the question of whether all proximal 3 parts PHF with a severely displaced GT are suitable for a full-percutaneous IMN technique remains unclear for the authors, due to the limited number of cases treated with this technique, short follow-up and the lack of a study comparing open and full-percutaneous IMN technique.

## Conclusion

The authors describe a novel technique for treating 3-part PHFs. Our article presents a departure from traditional methods, opting for a full-percutaneous reduction and IMN technique as an alternative to the commonly used approaches, and it is therefore beneficial as it requires only small incisions. We believe that this is might be a reliable method to treat the majority of 3-part PHFs but being familiar with the IMN surgical technique might be critical to avoid complications.

## Disclaimers:

Funding: No funding was disclosed by the authors.

Conflicts of interest: Marc-Olivier Gauci is a consultant for Stryker and NewclipTechnic. The other authors, their immediate families, and any research foundations with which they are affiliated have not received any financial payments or other benefits from any commercial entity related to the subject of this article.
